# Effect of dignity therapy on meaning in life scores of cancer patients in palliative care

**DOI:** 10.1017/S147895152510045X

**Published:** 2025-09-12

**Authors:** Michelle Uchida Miwa, Carlos Eduardo Paiva, Ana Julia Sucupira Ferreira, Miguel Julião, Harvey Max Chochinov, Welinton Yoshio Hirai, Ricardo dos Reis, Bianca Sakamoto Ribeiro Paiva

**Affiliations:** 1 Palliative Care Unit, Barretos Cancer Hospital, Barretos, São Paulo, Brazil; 2 Research Group on Palliative Care and Health-Related Quality of Life, Barretos Cancer Hospital, Barretos, São Paulo, Brazil; 3 Department of Clinical Oncology, Breast and Gynecology Division, Barretos Cancer Hospital, Barretos, São Paulo, Brazil; 4 Cuidados Paliativos, Equipa Comunitária de Suporte em Cuidados Paliativos de Sintra, Sintra, Portugal; 5 Department of Psychiatry, Max Rady College of Medicine, University of Manitoba, CancerCare Manitoba Research Institute, Winnipeg, MB, Canada; 6 Department of Epidemiology and Bioestatistics, Barretos Cancer Hospital, Barretos, São Paulo, Brazil; 7 Department of Gynecological Oncology, Barretos Cancer Hospital, São Paulo, Brazil

**Keywords:** dignity therapy, meaning in life, palliative care, spiritual well-being, emotional symptoms

## Abstract

**Objectives:**

Dignity Therapy (DT) helps reframe and give meaning to the illness process of the terminally ill individual. This study aims to evaluate the effect of DT on meaning in life scores and, additionally, to assess how much DT can alleviate physical and emotional symptoms in cancer patients undergoing palliative care.

**Methods:**

This was a before-and-after clinical trial, involving the recruitment of 30 patients hospitalized in a palliative care unit, who filled out the Edmonton Symptom Assessment Scale (ESAS) and the Meaning in Life Scale (MiLS) both before and after the implementation of DT.

**Results:**

Of the 40 patients invited to participate in the study, DT was completed by 30 (75%) participants: 22 (73%) women and 8 (27%) men. Eighteen (60%) patients died during hospitalization, while 12 (40%) were discharged. When analyzing the factors correlated with the MiLS scores, a positive association was identified between the emotional and physical domains of the ESAS, and a negative association with the total ESAS score, spiritual ESAS score, male gender, higher educational level, and a cancer diagnosis duration (>6 years).

**Significance of results:**

DT contributed to clinically relevant improvement, albeit not statistically significant, observed in emotional and spiritual well-being, as well as in the meaning of life. This underscores the importance of considering DT for palliative care patients nearing death.

## Introduction

The experience of life-threatening disease can generate a significant impact on patients’ mental health. Feelings such as insecurity, anxiety, worry, fear, loneliness, and anger arise, driven by the uncertainty of future events and a loss of sense of control in daily life. When such feelings and emotions are not managed, individuals can become overwhelmed, experiencing intense reactions such as panic attacks and burnout (NHS 2024; Robinson et al. 2019).

As illness progresses and functional capacity decreases, patients begin to experience various losses, impinging on their quality of life (Antoniadis et al. 2024; Moreira et al. 2021; Verkissen et al. 2019). The provision of palliative care becomes paramount, offering a holistic approach (Davis and Hui 2017; WHO 2020). This is also necessary to address “total pain” – a concept introduced by Dame Cicely Saunders – which describes how pain can derive from physical, emotional, social, and spiritual dimensions of patient experience (Clark 1999). Therefore, it is necessary to use a biopsychosocial model of care as opposed to the traditional biomedical model focusing exclusively on physical aspects of illness (Krawczyk et al. 2018).

Emotional states influence spiritual well-being, and vice versa (Beaussant et al. 2021; Siddall et al. 2015; Winger et al. 2021). Hence, coping mechanisms enable the reevaluation of priorities and adaptations in order to find new sources of meaning and purpose in life, providing the individual with resources to reinterpret their suffering through the convergence of values and beliefs and, consequently, the adjustment of resilience (Klikovac and Djurdjevic 2010).

Chochinov et al. developed the Model of Dignity in the Terminally Ill, which details elements subsumed under the rubric of dignity toward the end of life; the primary model themes relate to concerns about illness, psychological, spiritual, and social factors (Chochinov 2022; Chochinov et al. 2002a). Based on this model, dignity therapy (DT) was created, which seeks to help patients with life-limiting illness to reframe and give meaning to their lives (Chochinov 2012; Chochinov et al. 2005).

This practice improves patients’ emotional well-being, quality of life, and family cohesion (Chochinov et al. 2011; Scarton et al. 2018). Studies confirm its effectiveness in reducing distress, depression, anxiety, and psychological distress, enhancing end-of-life experience (Iani et al. 2020; Julião et al. 2014, 2017). Additionally, it increases dignity, satisfaction, peace, and meaning at the end of life (Donato et al. 2016; Li et al. 2020; Zaki-Nejad et al. 2020; Chen et al. 2021).

Although some studies mention the importance of meaning in life for patients at the end of life and the increase in their sense occurs through DT, which indicates a potential overlap with the concepts measured by Krause’s Meaning in Life Scale (MiLS), to the best of our knowledge, its impact on the dimensions of meaning and purpose in life, which this author divides into 4 main aspects: objective, purpose, values, and reflections, has not yet been registered (Krause 2009; Rabow 2019). Given the growing evidence of the relevance of this topic and the great usefulness and acceptance of DT intervention in the context of palliative care, this study aims to evaluate the effect of DT on meaning in life scores and additionally assess how much DT can alleviate physical and emotional symptoms in cancer patients undergoing palliative care.

## Methods

### Study design

This was a before-and-after clinical trial (Aggarwal and Ranganathan 2019) (registered in the Brazilian Registry of Clinical Trials (REBEC) under the number RBR-2nygd6j).

### Study setting

The study was conducted in the palliative care inpatient unit at Barretos Cancer Hospital (Barretos, São Paulo, Brazil).

### Participants

Patients were recruited if they met the following inclusion criteria: age ≥ 18 years, incurable disease, exclusive or non-exclusive follow-up with a palliative care team, knowledge of the illness itself (terminal prognosis), and ability to express oneself verbally and in writing in the Portuguese language spoken in Brazil. Individuals with psychiatric illnesses (dementia, personality, mood, depression, anxiety and psychotic disorders, and impaired cognitive ability) were excluded.

### Data collection and intervention

Participants were screened for eligibility by the researcher who is an experienced palliative care physician and is involved in hospitalized palliative patient care and had with prior training in DT. After providing written consent, participants completed a questionnaire that included age, gender, religion, educational level, marital status, number of children, main caregiver, diagnosis, time since diagnosis, and Palliative Performance Scale (PPS) (Maciel and Carvalho 2009).

### Instruments

The Edmonton Symptom Assessment System (ESAS-BR) consists of a brief scale focused on evaluating the intensity of symptoms through a gradation between 0 (minimum intensity) and 10 (maximum intensity) reported by the patient. Symptoms addressed include fatigue, nausea, depression, anxiety, drowsiness, appetite, well-being, dyspnea, quality of sleep, inner peace, and spiritual pain. This instrument has been validated for use in Brazil (Paiva et al. 2015).

The Portuguese translation of the MiLS consists of 8 questions with 4 subscales: values, purpose, objectives, and reflections. Values focus on beliefs and life philosophy; purpose addresses the search for meaning in life and how fulfilled individuals feel; goals concern personal objectives, sense of direction, and life goals; and reflections consider opinions regarding past choices and the level of peace individuals feel about these decisions. Likert responses are as follows: 1: I strongly disagree, 2: I disagree, 3: I agree, and 4: I very much agree. The score ranges from 8 to 32, with higher scores indicating greater meaning in life (Gravier et al. 2020). Within 24 h of applying these instruments, DT was performed; no more than a day later, these instruments were reapplied.

DT involves a brief interview guided by the Dignity Therapy Question Protocol, which has been translated and adapted into Brazilian Portuguese (Miwa *et al*., 2023). These interviews were audio recorded, transcribed verbatim, edited, and presented to the participants for approval before completing their legacy documents. While DT was conducted by the palliative care physician involved in recruitments, different researchers administered the pre- and post-measures.

### Ethical approval

This study was approved by the Committee of Ethics in Research of Barretos Cancer Hospital, no. 5.567.892/2022. All the participants invited to participate in the study signed an informed consent form.

### Statistical analysis

Study data were managed using REDCap electronic data capture tools (Harris et al. 2009), hosted at Barretos Cancer Hospital, and analyzed using R software version 4.4.0 2024.

To describe the sample, frequency and proportions were reported for qualitative variables; and range, medians, means, and standard deviations were reported for quantitative variables. Graphs were generated using the ggplot2 library in the R language. For the comparison between ESAS and MiLS scores in the pre- and post-conditions, the non-parametric Wilcoxon test was employed due to the non-normality of the scores. To analyze associations between factors (covariates) and the response variable (outcome), both simple and multiple linear regression models were used. The selection of significant covariates for the multiple models was performed using the Backward method. The multivariate analysis presented a limitation due to the multiple models containing too many factors for a dataset with 30 cases. Therefore, prior to applying the backward method, the variables “number of children,” “primary caregiver,” and “diagnosis (neoplasms)” were excluded.

To analyze the ESAS aspects separately before and after the intervention, the global domains were grouped into 3 categories: emotional (including depression and anxiety), with a score ranging between 0 and 20, physical (encompassing pain, fatigue, nausea, drowsiness, appetite, dyspnea, and sleep quality), with a score ranging between 0 and 70, and emotional (including inner peace and spiritual pain), with a score ranging between 0 and 20. It is noteworthy that on this scale, the higher the score given, the worse the symptom. Similarly, for the MiLS, the analysis focused on its 4 dimensions: values, purposes, objectives, and reflections.

## Results

Four hundred patients were screen, 360 who did not meet eligibility criteria due to the end of life (83; 22.9%), inadequate personal coping (69; 19.1%), delirium (71; 19.6%), uncontrolled symptoms (44; 12.1%), impaired cognition (40; 11%), contact isolation (41; 11.3%), inadequate family coping (16; 4.4%), hospitalization for less than 7 days (16; 4.4%), social issues (10; 2.7%), didn’t speak Portuguese (7; 1.9%), lack of knowledge of the prognosis (5; 1.3%), psychiatric disorder (4; 1.1%), and impaired diction (3; 0.08%).

**Figure 1.**
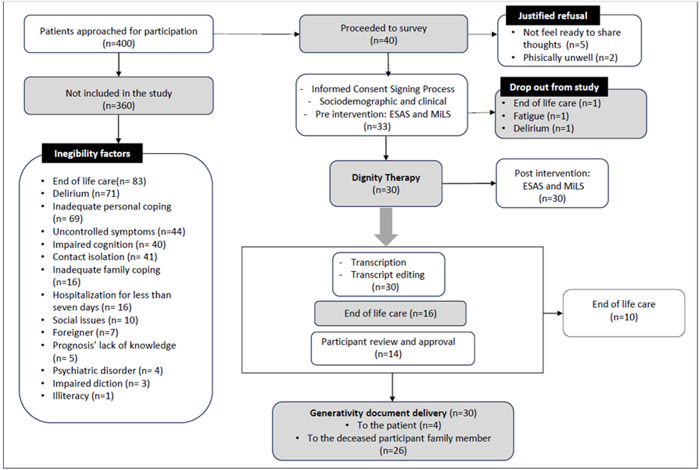
Patient flowchart in the study.

Forty patients were invited to participate. Of those, 7 (17.5%) declined, either because they did not feel ready to share their thoughts or were physically unwell. Additionally, 3 (7%) withdrew during the study due to worsening symptoms that prevented them from completing the interview. Thirty patients completed DT; 22 (73%) females and 8 (27%) males. Eighteen (60%) passed away during hospitalization, while 12 (40%) were discharged. Of the 30 legacy documents, 16 (53.3%) were finalized without participant final approval due to significant clinical decline that prevented them from reviewing the edited documents. Of the 14 (46.6%) participants who approved their documents, 10 (33%) progressed to end-of-life care. Ultimately, 26 (86.6%) legacy documents were delivered to participants’ families (Figure 1). The social and clinical characteristics of patients are presented in Table 1.

**Table 1.** Social and clinical characteristics of the participants

Variable			Participants	
			*N*	%
Sex	Female		22	73
	Male		8	27
Marital status	Single		7	23
	Married/stable union		16	53.3
	Divorced		5	17
	Widower		2	6.7
Number of children	None		7	23
	1		5	17
	2		5	17
	3		9	30
	≥4		4	13
Education	Primary		7	23
	Secondary		14	46.6
	Higher		9	30
Religion	Catholic		15	52
	Evangelical		8	28
	Spiritist		5	17
	Other^a^		1	3.4
	Ignored		1	3.4
Primary caregiver	Spouse		8	27
	Children		6	20
	Brother/Sister		5	16
	Parents		8	27
	Other^b^		3	10
Diagnosis (neoplasms)	Gastrointestinal tract		6	20
	Lung		6	20
	Breast		6	20
	Genitourinary		6	20
	Sarcomas		2	6.7
	Other^c^		4	13
Time since diagnosis (years)	<1		8	27
	1 to 5		15	50
	>6		7	10
PPS	30		2	6.7
	40		7	23
	50		13	43
	60		4	13
	70		3	10
	80		1	3.3
ECOG	1		4	13
	2		17	57
	3		9	30
Age	Minimum	Median	Mean (SD)	Maximum
	27	53	52 (13)	83

PPS: Palliative Performance Scale.

a Other: Seicho-no-Ie.

b Other: friend, son-in-law, and formal caregiver.

c Other: amygdala, parotid, submandibular, and chordoma.

The DT interviews lasted an average of 50 min (45–80), with the shortest being 45 mi and the longest lasting 80 min. The tone of the interviews was relaxed, aiming to make participants as comfortable as possible so they could freely share their thoughts. As outlined in the methodology, the question protocol was presented to participants 24 h before the interview. During the interviews, participants were encouraged to avoid discussing uncomfortable memories or those that could cause embarrassment. This approach prompted participants to reflect and, in some cases, choose not to include certain sections. Additionally, there was no need for follow-up support from the psychology team after the DT sessions, as no significant emotional distress was triggered by the intervention.

We calculated Cronbach’s alpha for both instruments used before and after DT. Cronbach’s alpha for ESAS was 0.87 and 0.831 before and after DT, respectively. For MiLS, Cronbach’s alpha was 0.732 and 0.776 before and after the intervention, respectively. These values suggest good reliability and that the instrument questions are highly correlated with each other.

**Figure 2.**
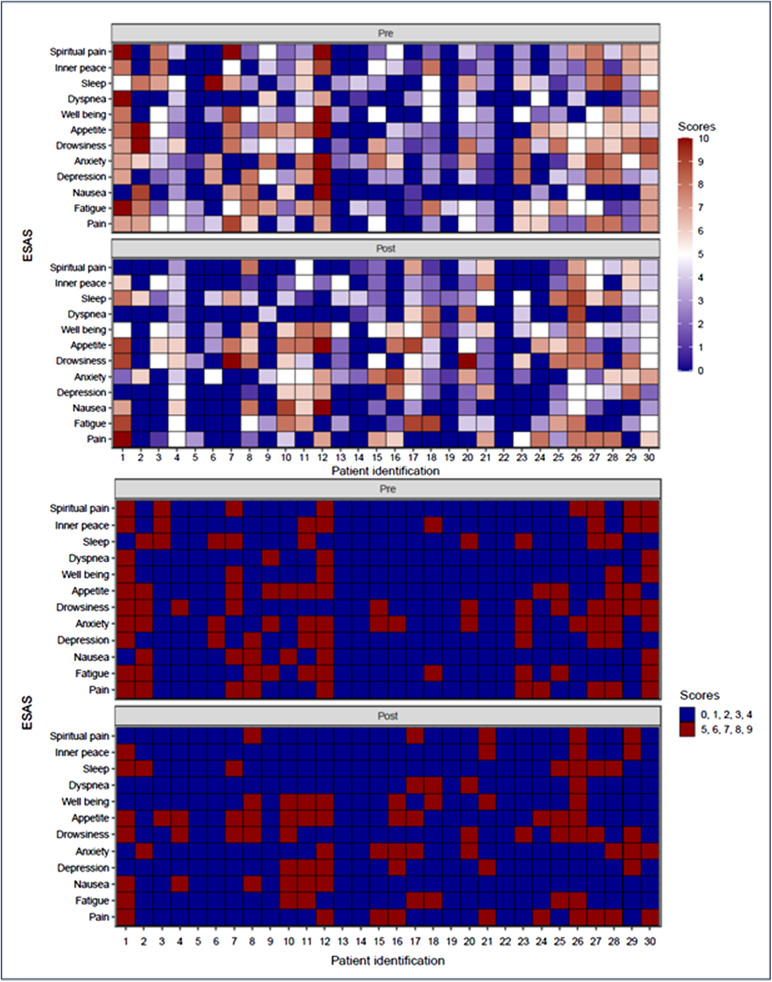
ESAS heatmap with analysis of individuals before and after DT intervention.

**Figure 3.**
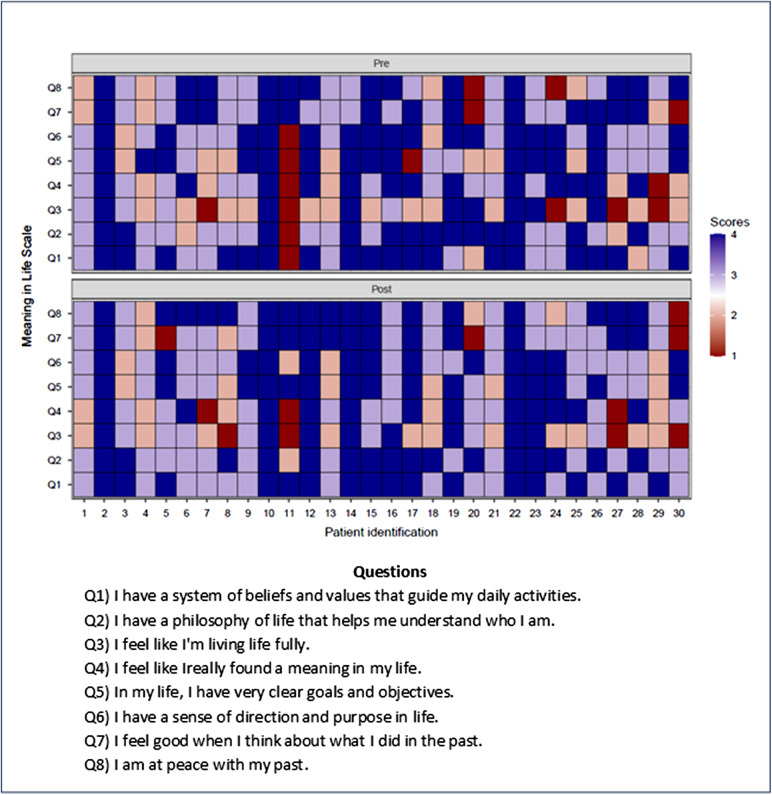
Meaning in Life Scale heatmap with analysis of individuals before and after DT intervention.

When comparing the heatmaps to the ESAS scale, a discreet trend towards symptom relief is observed, that is, some areas that were previously predominantly red show a slightly lighter color, which may suggest a tendency, although modest, positive in the reduction of symptoms. The same is true for the MiLS (Figures 2 and 3).

### ESAS

No statistically significant before-and-after-intervention differences were found between emotional (*p* = 0.3), physical (*p* = 0.6), and spiritual (*p* = 0.2) domains. Within the emotional domain, the mean (standard deviation [SD]) shifted from 7.8 (6) at before intervention to 6 (4.9) after intervention, while in the physical domain it shifted from 25 (14) to 22 (14) and in the spiritual domain it shifted from 6.7 (6.1) to 4.5 (4.6) (Table 2).

**Table 2.** Comparison between ESAS and MiLS pre- and post-intervention

ESAS, minimum/median/maximum mean (SD)	Pre	Post	*p*-value^a^
Emotional domain^b^	0/7/20	0/5.5/17	0.3
	7.8 (6)	6.0 (4.9)	
Physical domain^c^	0/26/52	0/20/55	0.6
	25 (14)	22 (14)	
Espiritual domain^d^	0/5.5/19	0/3.5/15	0,2
	6.7 (6.1)	4.5 (4.6)	
Total^e^	0/46/91	0/41/88	0.4
	43 (24)	37 (21)	
MiLS, minimum/median/maximum mean (SD)			
Values subscore	2/7/8	2/7/8	0.5
	6.77 (1.28)	7.03 (0.81)	
Purposes subscore	2/6/8	2/6/8	0.9
	5.63 (1.77)	5.67 (1.84)	
Goals subscore	2/6.5/8	4/6.5/8	0.9
	6.5 (1.59)	6.6 (1.38)	
Reflections subscore	2/6/8	2/6.5/8	0.6
	6.37 (1.56)	6.53 (1.59)	
Total	14/25/32	20/26/32	0.8
	25.3 (4.2)	25.8 (4.1)	

ESAS: Edmonton Symptom Assessment System; MiLS: Meaning in Life Scale; SD: Standard Deviation.

a *p*-value: Wilcoxon rank sum test.

b Emotional domain: depression and anxiety.

c Physical domain: pain, fatigue, nausea, drowsiness, appetite, dyspnea, and sleep.

d Spiritual domain: spiritual pain and inner peace.

e Total: emotional domain, physical domain, spiritual domain, and well-being.

### MiLS

There were no statistically significant differences before and after DT between the 4 dimensions: values (*p* = 0.5), purpose (*p* = 0.9), objectives (*p* = 0.9), and reflections (*p* = 0.6). The mean for values shifted from 6.77(1.28) at pre-intervention to 7.03 (0.81), while in the purpose dimension, it shifted from 5.63 (1.77) to 5.67 (1.84); the mean for objectives shifted from 6.5 (1.59) at pre-intervention to 6.6 (1.38), while reflection shifted from 6.37 (1.56) to 6.53 (1.59) (Table 2).

### Predictors of meaning in life

To examine the relationship between MiLS and other variables, both univariate and multivariate statistical evaluations were performed, considering the mean scores from pre- and post-intervention for the ESAS domains. The univariate and multivariate statistical evaluations referred to the total MiLS score, aiming to identify potential relationships and significant variations among the studied variables. (Results for the subscores [values, purposes, goals, and reflections] are available in the Supplementary material.)

In the univariate analysis, significant negative associations were found between ESAS spiritual domain (*p* = 0.003), ESAS total domain (*p* = 0.024), higher level education (*p* = 0.045), and being single compared to being married/stable union (*p* = 0.01). On the other hand, being evangelical was associated with a greater meaning in life relative to the catholic religion (*p* = 0.046). Other demographic variables such as age, gender, time since cancer diagnosis, and PPS, as well as ESAS emotional and physical domains, were not associated with meaning in life scores (Table 3).

**Table 3.** Factors associated with meaning in life total score (mean)

	Meaning in life scale total score (mean)			
	Univariate estimate (95% CI)	*p*-value	Multivariate estimate (95% CI)	*p*-value
Emotional domain^a^	−0.09 (−0.42, 0.24)	0.6	1.4 (0.34, 2.4)	**0.012**
Physical domain^b^	−0.11 (−0.23, 0.01)	0.060	1 (0.17, 1.9)	**0.022**
Spiritual domain^c^	−0.48 (−0.78, −0.17)	**0.003**	0.61 (−0.44, 1.7)	0.2
Total domain^d^	−0.08 (−0.16, 0.01)	**0.024**	−1 (−1.8, −0.16)	**0.022**
Age	0.09 (−0.02, 0.20)	0.11		
Gender				
Female	–		–	
Male	−1.8 (−5.1, 1.6)	0.3	−3.1 (−5.9, −0.26)	**0.034**
Religion				
Catholic	–			
Spiritist	3.2 (−0.47, 6.9)	0.085		
Evangelical	3.4 (0.06, 6.7)	**0.046**		
Education				
Primary	–		–	
Secondary	−2.8 (−6.4, 0.79)	0.12	−2.4 (−5.6, 0.72)	0.12
Higher	−4.0 (−7.9, −0.09)	**0.045**	−2.5 (−6.6, 1.5)	0.2
Marital status				
Married/stable union	–		–	
Divorced	−1.5 (−5.3, 2.3)	0.4	−2.3 (−5.7, 1.2)	0.2
Single	−4.6 (−7.9, −1.2)	**0.01**	−2.8 (−6.0, 0.38)	0.08
Widower	1.2 (−4.4, 6.8)	0.7	1.1 (−4.1, 6.2)	0.7
Time since diagnosis (years)				
< 1	–		–	
1 and 5	−0.75 (−4.4, 2.9)	0.7	−0.9 (−3.5, 1.7)	0.5
>6	−1.7 (−6.0, 2.6)	0.4	−4.3 (−7.6, −0.97)	**0.015**
PPS				
30%	–			
40%	−4.3 (−11, 2.1)	0.2		
60%–50%	−1.8 (−7.7, 4.2)	0.5		
80%–70%	0.38 (−6.5, 7.2)	>0.9		

PPS: Palliative Performance Scale; CI: confidence interval.

a Emotional domain: depression and anxiety.

b Physical domain: pain, fatigue, nausea, drowsiness, appetite, dyspnea, and sleep.

c Espiritual domain: spiritual pain and inner peace.

d Total: emotional domain, physical domain, spiritual domain, and well-being.

In the multivariate regression, independent factors were positively associated with meaning in life. These included ESAS emotional (*p* = 0.012) and physical (*p* = 0.022) domains. In contrast, significant negative associations were found between meaning in life and ESAS total domain (*p* = 0.022), male gender (*p* = 0.034), and time since cancer diagnosis greater than 6 years (*p* = 0.015) (Table 3).

Considering that the mean scores obtained in the pre- and post-intervention periods were very similar, univariate and multivariate regression analyses were also performed for both pre- and post-intervention time points (Table 4).

**Table 4.** Factors associated with meaning in life total score (pre- and post-intervention)

Meaning in life scale total score (pre- and post-intervention)								
	Pre-intervention				Post-intervention			
	Univariate estimate (95% CI)	*p*-value	Multivariate estimate (95% CI)	*p*-value	Univariate estimate (95% CI)	*p*-value	Multivariate estimate (95% CI)	*p*-value
Emotional domain^a^	−0.14 (−0.40, 0.13)	0.3	1.1 (0.01, 2.2)	**0.048**	0.01 (−0.31, 0.33)	>0.9	0.33 (0.07, 0.59)	**0.016**
Physical domain^b^	−0.08 (−0.19, 0.04)	0.2	1.1 (0.14, 2.1)	**0.028**	−0.1 (−0.21, 0.0)	0.059	0.06 (−0.06, 0.17)	0.3
Spiritual domain^c^	−0.22 (−0.48, 0.03)	0.085	1.1 (−0.08, 2.2)	0.066	−0.44 (−0.74, −0.15)	**0.005**	−0.48 (−0.79, −0.17)	**0.005**
Total domain^d^	−0.06 (−0.12, 0.0)	0.068	−1.1 (−2.0, −0.15)	**0.026**	−0.07 (−0.14, 0.0)	**0.045**	–	–
Age	0.08 (−0.04, 0.2)	0.2	−0.11 (−0.29, 0.07)	0.2	0.1 (−0.02, 0.21)	0.09	0.06 (−0.04, 0.16)	0.2
Gender								
Female	–	–	–	–	–	–	–	–
Male	−0.88 (−4.5, 2.7)	0.6	−2.5 (−6.4, 1.4)	0.2	−2.7 (−6.0, 0.68)	0.11	−4.2 (−6.9, −1.5)	**0.005**
Religion								
Catholic	–	–	–	–	–	–	–	–
Spiritist	3.2 (−0.77, 7.2)	0.11	–	–	3.2 (−0.59, 6.9)	0.1	–	–
Evangelical	3.0 (−0.6, 6.7)	0.1	–	–	3.8 (0.34, 7.2)	**0.032**	–	–
Education								
Primary	–	–	–	–	–	–	–	–
Secondary	−3.3 (−7.1, 0.57)	0.092	−4.4 (−8.8, −0.05)	**0.048**	−2.4 (−6.1, 1.4)	0.2	–	–
Higher	−4.0 (−8.2, 0.2)	0.061	−5.3 (−11, 0.63)	0.075	−4.0 (−8.1, 0.03)	0.051	–	–
Marital status								
Married/Stable union	–	–	–	–	–	–	–	–
Divorced	−2.0 (−6.1, 2.0)	0.3	−5.5 (−10, −0.12)	**0.046**	−0.94 (−4.9, 3.1)	0.6	−0.9 (−4.3, 2.5)	0.6
Single	−4.8 (−8.4, −1.2)	**0.011**	−4.3 (−8.7, 0.17)	0.058	−4.4 (−7.9, −0.82)	**0.018**	−4.3 (−7.3, −1.2)	**0.01**
Widower	1.4 (−4.6, 7.3)	0.6	1.1 (−7.2, 9.3)	0.8	1.1 (−4.8, 6.9)	0.7	−1.6 (−6.6, 3.4)	0.5
Time since diagnosis (years)								
<1	–	–	–	–	–	–	–	–
1 and 5	−0.34 (−4.2, 3.5)	0.9	−1.8 (−5.8, 2.2)	0.3	−1.2 (−4.9, 2.6)	0.5	−1.0 (−3.6, 1.5)	0.4
>6	−1.9 (−6.4, 2.7)	0.4	−5.6 (−10, −0.79)	**0.026**	−1.5 (−5.9, 3.0)	0.5	−3.8 (−7.2, −0.45)	**0.029**
PPS								
30%	–	–	–	–	–	–	–	–
40%	−4.7 (−12, 2.2)	0.2	−0.42 (−8.3, 7.5)	>0.9	−3.9 (−10, 2.6)	0.2	−5.2 (−10, −0.47)	**0.034**
60%–50%	−2.7 (−9.2, 3.7)	0.4	−2.0 (−9.1, 5.1)	0.6	−0.82 (−6.8, 5.2)	0.8	−3.7 (−8.0, 0.71)	0.094
80%–70%	−0.75 (−8.2, 6.7)	0.8	1.5 (−7.7, 11)	0.7	1.5 (−5.5, 8.5)	0.7	0.0 (−5.2, 5.2)	>0.9

PPS: Palliative Performance Scale; CI: confidence interval.

a Emotional domain: depression and anxiety.

b Physical domain: pain, fatigue, nausea, drowsiness, appetite, dyspnea, and sleep.

c Espiritual domain: spiritual pain and inner peace.

d Total: emotional domain, physical domain, spiritual domain, and well-being.

In the pre-intervention univariate analysis, a significant negative association was found between being single compared to being married/stable union (*p* = 0.01). In contrast, post DT, significant negative correlations were found for the ESAS spiritual domain (*p* = 0.005), the total ESAS score (*p* = 0.045), and being single compared to being married/stable union (*p* = 0.018). On the other hand, being evangelical was associated with a greater meaning in life compared to the catholic religion (*p* = 0.046) in the post-intervention period.

In the multivariate regression prior to DT, independent factors were positively associated with meaning in life, including the ESAS emotional (*p* = 0.048) and physical (*p* = 0.028) domains. In contrast, significant negative associations were found for the total ESAS domain (*p* = 0.026), secondary education level (*p* = 0.048), being divorced (*p* = 0.046), and a time since cancer diagnosis greater than 6 years (*p* = 0.026). After DT, significant negative correlations were found for the ESAS spiritual domain (*p* = 0.005), male gender (*p* = 0.005), being single (*p* = 0.01), KPS 40% (*p* = 0.034), and time since cancer diagnosis greater than 6 years (*p* = 0.029). The ESAS emotional domain maintained a significant positive correlation (*p* = 0.016).

## Discussion

This is an original study conducted in Brazil, aimed at evaluating the effects of DT using the MiLS. We also examined the impact of this intervention on the physical and emotional aspects of cancer patients undergoing palliative care. The analyses demonstrated a trend toward improvement in both the meaning of life scores and the assessment of symptoms through ESAS after DT. In addition, there is a strong correlation between the intensity of symptoms (physical, emotional, and spiritual), gender, educational level, marital status, and time since cancer diagnosis with the individual’s meaning of life. Although early referral to palliative care offers greater benefits (WHO 2020), most patients are referred late, often in their final months or days. This delay stems from myths about palliative care and challenges healthcare teams face in timing the transition. As a result, recruitment and follow-up in research are hindered by patients’ increasing frailty and declining functional capacity, including concentration (Addington-Hall 2009; Higginson 2016). Additionally, the psychosocial and spiritual impacts of life-threatening conditions may further limit research participation (Addington-Hall 2009; Ramos et al. 2024).

Given the progression of the disease and the consequent alteration of self-image, increased dependency, exacerbation of symptoms, and recurrent hospitalizations, the ill individual experiences a period of uncertainty and fear, which may culminate in the loss of dignity, potentially leading to a desire to die or a loss of the will to live (Chochinov et al. 2002b, 2002a). Thus, when assessing the effect of DT on the emotional (anxiety and depression), physical (pain, fatigue, nausea, drowsiness, appetite, dyspnea, and sleep quality), and spiritual (spiritual pain and inner peace) domains of the ESAS through the heatmap, a general trend of improvement is observed (Figure 2).

Our analysis reveals a greater tendency for improvement after DT in the emotional and spiritual domains compared to the physical ones, with anxiety showing greater relief than depression. Although studies have shown improvement in anxiety with DT, the data on depression are less consistent (Chochinov et al. 2011; Martínez et al. 2017). Deng et al. (2025) and Cuevas et al. (2021) report improvements in depression levels following DT, while Julião et al. (2014) demonstrated such improvements, but observed a decline in depression on the fourth and thirtieth days, with no change on the fifteenth day after the intervention. In contrast, Houmann et al. (2014) and Rudilla et al. (2016) reported a significant negative effect of DT on depression.

In relation to the impact of the DT on the spiritual domain, the findings are consistent with the literature, tending toward improvement and/or maintenance of inner peace at the end of life (Austin et al. 2025; Iani et al. 2020; Vincenzo et al. 2023). Elias et al. (2008) identified 6 categories and 11 subcategories related to the nature of spiritual pain, including the fear of being forgotten and disintegration. DT can address spiritual pain through the creation of legacy, which aims to immortalize the patient’s existence (Chochinov et al. 2005).

A slight improvement in drowsiness and fatigue was observed, with appetite worsening slightly and no changes in dyspnea, nausea, or pain. Since DT does not directly target physical symptoms, these outcomes are unsurprising (Hall et al. 2012; Scarton et al. 2018). However, DT may mitigate drowsiness and fatigue by motivating patients to engage in life story narration and interaction with the therapist (Julião et al. 2014).

Concerning to the heatmap, which illustrates the meaning of life before and after the intervention, it was identified a general trend of improvement in the subscores related to values, purposes, goals, and reflections. These findings suggest that the intervention had a positive impact on participants’ sense of meaning in life, as both the heatmap and subscores showed an increased perception of meaning, which may indicate an improvement in sense of dignity and consequently in the quality of life.

To the best of our knowledge, there are no studies that assess the meaning of life and its relationship with DT. Our findings support the association identified by Liu et al. (2021) and Oh and Shin (2014), who found that higher levels of meaning in life are directly proportional to an individual’s sense of dignity. They report that individuals with a high sense of meaning in life are at lower risk of losing their sense of dignity. This suggests a role for interventions aimed at dignity, helping patients reflect and integrate key facets of their lives, thereby alleviating the psychological suffering that the illness process may induce (Oh and Shin 2014).

The results of both uni- and multivariate regression analyses indicate that several factors are associated with higher MiLS scores, including religious affiliation (particularly being evangelical), which fosters meaning through rituals and traditions (Dilmaghani 2018; Fletcher 2004; Krause 2009; Rizvi and Hossain 2017). Lower scores on the spiritual (spiritual pain and inner peace) and total (emotional, physical, spiritual ESAS domains, and well-being) domains of the ESAS were associated with a greater sense of meaning in life. Conversely, lower MiLS scores were linked to higher levels of education, being male, being single, and longer diagnosis duration (>6 years), with prolonged exposure to loss of control and uncertainty about the future contributing to reduced quality of life and resilience (Rodriguez-Gonzalez et al. 2022).

Interestingly, the positive correlation between the emotional and physical ESAS domains and MiLS aligns with Viktor Frankl’s existential perspective, which proposes that suffering can act as a catalyst for the search for meaning in life. Although one might expect the intensification of symptoms would decrease meaning, Frankl’s concept of attitudinal values emphasizes the power of choosing one’s response to suffering, aligning with the idea that individuals facing significant physical and emotional suffering may find meaning in their experiences by reevaluating their lives and the suffering they endure (Frankl 1991; Moreira and Holanda 2010; Silveira and Gradim 2015).

This process of finding meaning through suffering is facilitated by interventions like DT, which promote existential reflection. DT, along with the pre- and post-intervention instruments, likely helped participants reflect on their experiences and find meaning despite their suffering.

This study has some limitations. The first one is the sample size, as well as the pharmacological interventions, which may have influenced symptom control and responses in the physical domain of the ESAS. The second is related to the fact that the study was conducted in a single healthcare center, exclusively with patients diagnosed with advanced cancer and limited life expectancy. However, since this center receives patients from all regions of Brazil, it may, to some extent, represent the cultural diversity of the country. The third concerns the positive correlation observed between intense emotional and physical symptoms and meaning in life, which may have been influenced by a potential regression dilution effect, as the pre- and post-intervention values showed minimal variation, likely due to data fluctuations. Finally, the main challenge faced was participant recruitment, as many did not meet eligibility criteria or declined participation due to emotional unpreparedness, physical discomfort, or rapid disease progression. Furthermore, several legacy documents could not be delivered, as participants entered the terminal phase before the study was completed. Edited legacy documents were shared with families as outlined in the Informed Consent Form.

Safeguarding dignity is essential in maintaining or bolstering meaning of life. This study showed a trend for DT to contribute to an improvement in emotional and spiritual well-being and enhanced the meaning of life. This highlights the importance of tracking meaning of life when providing and studying DT. To further deepen the understanding of these findings, future randomized, multicenter clinical trials are required.

## Supporting information

10.1017/S147895152510045X.sm001 Uchida Miwa et al. supplementary material Uchida Miwa et al. supplementary material

## Data Availability

All data relevant to the study are included in the article or uploaded as supplementary information.

## Supplementary material

The supplementary material for this article can be found at https://doi.org/10.1017/S147895152510045X.

## References

[ref1] Addington-Hall J (2009) The challenges of palliative care research. In *Research methods in palliative care*, 1–9.

[ref2] Aggarwal R and Ranganathan P (2019) Study designs: part 4 – interventional studies. *Perspectives in Clinical Research* 10(3), 137–139. doi:10.4103/picr.PICR_91_19 31404185 PMC6647894

[ref3] Antoniadis D, Giakoustidis A, Papadopoulos V, et al. (2024) Quality of life, distress and psychological adjustment in patients with colon cancer. *European Journal of Oncology Nursing* 68, 102467. doi:10.1016/j.ejon.2023.102467 38006715

[ref4] Austin PD, Lee W, Keall R, et al. (2025) Efficacy of spiritual interventions in palliative care: An umbrella review of systematic reviews. *Palliative Medicine* 39(1), 70–85. doi:10.1177/02692163241287650 39412883 PMC11673315

[ref5] Beaussant Y, Nichipor A and Balboni TA (2021) Integration of spiritual care into palliative care service delivery models. In Cherny, Nathan I., and others (eds), *Oxford Textbook of Palliative Medicine*. 6th edn. Oxford University Press, 1072–1079.

[ref6] Chen J, Yan, J, C, Wang, et al. (2021) Effects and satisfaction of dignity therapy among patients with hematologic neoplasms in the Chinese cultural context: a randomized controlled trial. *Supportive Care in Cancer: Official Journal of the Multinational Association of Supportive Care in Cancer* 29(11), 6819–6829. doi: 10.1007/s00520-021-06227-4 33999270

[ref7] Chochinov H (2012) Introducing dignity therapy to patients and families. In *Dignity Therapy: Final Words for Final Days*. Oxford University Press, 54–73.

[ref8] Chochinov H (2022) The model in detail. https://www.dignityincare.ca/en/the-model-in-detail.html (accessed 13 October 2024).

[ref9] Chochinov H, Hack T, McClement S, et al. (2002a) Dignity in the terminally ill: A developing empirical model. *Social Science & Medicine (1982)* 54(3), 433–443. doi:10.1016/s0277-9536(01)00084-3 11824919

[ref10] Chochinov HM, Hack T, Hassard T, et al. (2002b) Dignity in the terminally ill: A cross-sectional, cohort study. *Lancet (London, England)* 360(9350), 2026–2030. doi:10.1016/S0140-6736(02)12022-8 12504398

[ref11] Chochinov HM, Hack T, Hassard T, et al. (2005) Dignity therapy: A novel psychotherapeutic intervention for patients near the end of life. *Journal of Clinical Oncology: Official Journal of the American Society of Clinical Oncology* 23(24), 5520–5525. doi:10.1200/JCO.2005.08.391 16110012

[ref12] Chochinov HM, Kristjanson LJ, Breitbart W, et al. (2011) Effect of dignity therapy on distress and end-of-life experience in terminally ill patients: A randomised controlled trial. *The Lancet Oncology* 12(8), 753–762. doi:10.1016/S1470-2045(11)70153-X 21741309 PMC3185066

[ref13] Clark D (1999) ‘Total pain,’ disciplinary power and the body in the work of Cicely Saunders, 1958-1967. *Social Science & Medicine (1982)* 49(6), 727–736. doi:10.1016/s0277-9536(99)00098-2 10459885

[ref14] Cuevas PE, Davidson P, Mejilla J, et al. (2021) Dignity therapy for end-of-life care patients: A Literature Review. *Journal of Patient Experience* 8, 2374373521996951. doi:10.1177/2374373521996951 34179373 PMC8205385

[ref15] Davis MP and Hui D (2017) Quality of Life in Palliative Care. *Expert Review of Quality of Life in Cancer Care* 2(6), 293–302. doi:10.1080/23809000.2017.1400911 30854466 PMC6405258

[ref16] Deng Y, Yao Y, Wang C, et al. (2025) Effects of dignity therapy on dignity, anxiety, depression and quality of life for people with burns: A randomised controlled trial. *Journal of Advanced Nursing* 81(5), 2722–2738. doi:10.1111/jan.16466 39304304

[ref17] Dilmaghani M (2018) Religiosity and subjective wellbeing in Canada. *Journal of Happiness Studies* 19(3), 629–647. doi:10.1007/s10902-016-9837-7

[ref18] Donato SCT, Matuoka JY, Yamashita CC, et al. (2016) Effects of dignity therapy on terminally ill patients: a systematic review. *Revista da Escola de Enfermagem da USP* 50(6), 1014–1024. doi: 10.1590/s0080-623420160000700019 28198968

[ref19] Elias ACA, Giglio JS and Pimenta CADM (2008) Análise da natureza da dor espiritual apresentada por pacientes terminais e o processo de sua re-significação através da intervenção relaxamento, imagens mentais e espiritualidade (RIME). *Revista Latino-Americana de Enfermagem* 16, 959–965. doi:10.1590/S0104-11692008000600004 19229397

[ref20] Fletcher SK (2004) Religion and life meaning: differentiating between religious beliefs and religious community in constructing life meaning. *Journal of Aging Studies* 18(2), 171–185. doi:10.1016/j.jaging.2004.01.005

[ref21] Frankl VE (1991) *Em Busca de Sentido*. 60th edn. Petrópolis: Vozes.

[ref22] Gravier AL, Shamieh O, Paiva CE, et al. (2020) Meaning in life in patients with advanced cancer: a multinational study. *Supportive Care in Cancer: Official Journal of the Multinational Association of Supportive Care in Cancer* 28(8), 3927–3934. doi:10.1007/s00520-019-05239-5 31858248 PMC8582319

[ref23] Hall S, Goddard C, Opio D, et al. (2012) Feasibility, acceptability and potential effectiveness of Dignity Therapy for older people in care homes: a phase II randomized controlled trial of a brief palliative care psychotherapy. *Palliative Medicine* 26(5), 703–712. doi: 10.1177/0269216311418145 21859743

[ref24] Harris PA, Taylor R, Thielke R, et al. (2009) Journal of biomedical informatics. *Research Electronic Data Capture (Redcap) a Metadata-Driven Methodology and Workflow Process for Providing Translational Research Informatics Support* 42, 377–381. 10.1016/j.jbi.2008.08.010 PMC2700030 18929686

[ref25] Higginson IJ (2016) Research challenges in palliative and end of life care. *BMJ Supportive & Palliative Care* 6(1), 2–4. doi: 10.1136/bmjspcare-2015-001091 PMC4789687 26893386

[ref26] Houmann LJ, Chochinov HM, Kristjanson LJ, et al. (2014) A prospective evaluation of Dignity Therapy in advanced cancer patients admitted to palliative care. *Palliative Medicine* 28(5), 448–458. doi:10.1177/0269216313514883 24311296

[ref27] Iani L, De Vincenzo F, Maruelli A, et al. (2020) Dignity therapy helps terminally ill patients maintain a sense of peace: Early results of a randomized controlled trial. *Frontiers in Psychology* 11, 1468. doi:10.3389/fpsyg.2020.01468 32670169 PMC7330164

[ref28] Julião M, Oliveira F, Nunes B, et al. (2014) Efficacy of dignity therapy on depression and anxiety in Portuguese terminally ill patients: A phase II randomized controlled trial. *Journal of Palliative Medicine* 17(6), 688–695. doi:10.1089/jpm.2013.0567 24735024

[ref29] Julião M, Oliveira F, Nunes B, et al. (2017) Effect of dignity therapy on end-of-life psychological distress in terminally ill Portuguese patients: A randomized controlled trial. *Palliative & Supportive Care* 15(6), 628–637. doi: 10.1017/S1478951516001140 28166861

[ref30] Klikovac T and Djurdjevic A (2010) Psychological aspects of the cancer patients’ education: thoughts, feelings, behavior and body reactions of patients faced with diagnosis of cancer. *Journal of BUON: Official Journal of the Balkan Union of Oncology* 15(1), 153–156. 20414944

[ref31] Krause N (2009) Meaning in Life and Mortality. *The Journals of Gerontology Series B, Psychological Sciences and Social Sciences* 64B(4), 517–527. doi:10.1093/geronb/gbp047 PMC2905132 19515991

[ref32] Krawczyk M, Wood J and Clark D (2018) Total pain: Origins, current practice, and future directions. *The Norwegian Journal of Palliative Care* 2018(2), 6–10.

[ref33] Li YC, Feng YH, Chiang HY, et al. (2020) The Effectiveness of Dignity Therapy as Applied to End-of-Life Patients with Cancer in Taiwan: A Quasi-Experimental Study. *Asian Nursing Research* 14(4), 189–195. doi: 10.1016/j.anr.2020.04.003 32335317

[ref34] Liu X, Liu Z, Cheng Q, et al. (2021) Effects of meaning in life and individual characteristics on dignity in patients with advanced cancer in China: A cross-sectional study. *Supportive Care in Cancer: Official Journal of the Multinational Association of Supportive Care in Cancer* 29(5), 2319–2326. doi:10.1007/s00520-020-05732-2 32914328

[ref35] Maciel MGS and Carvalho RT (2009) Portuguese (Brazilian) translation of the Palliative Performance Scale (PPSv2). Victoria Hospice Society. https://victoriahospice.org/wp-content/uploads/2019/07/pps_-_portuguese_brazilian_-_sample.pdf.

[ref36] Martínez M, Arantzamendi M, Belar A, et al. (2017) ‘Dignity therapy,’ a promising intervention in palliative care: A comprehensive systematic literature review. *Palliative Medicine* 31(6), 492–509. doi:10.1177/0269216316665562 27566756 PMC5405836

[ref37] Miwa MU, Paiva CE, Ferreira AJS, et al. (2023) Translation and cross-cultural adaptation of the Dignity Therapy Question Protocol to Brazilian Portuguese. *Palliative and Supportive Care* 21(856), 62. 10.1017/S147895152300041X 37052333

[ref38] Moreira DP, Simino GPR, Reis IA, et al. (2021) Quality of life of patients with cancer undergoing chemotherapy in hospitals in Belo Horizonte, Minas Gerais State, Brazil: does individual characteristics matter? *Cadernos de Saúde Pública* 37(8), e00002220. doi:10.1590/0102-311x00002220 34550177

[ref39] Moreira N and Holanda A (2010) Logoterapia e o sentido do sofrimento: convergências nas dimensões espiritual e religiosa. *Psico-USF* 15, 345–356. doi:10.1590/S1413-82712010000300008

[ref40] NHS (2024) Emotional effects of an illness or condition. https://www.nhsinform.scot/care-support-and-rights/palliative-care/mental-health-and-wellbeing/emotional-effects-of-an-illness-or-condition/ (accessed 13 October 2024).

[ref41] Oh PJ and Shin SR (2014) Effects of dignity interventions on psychosocial and existential distress in terminally ill patients: a meta-analysis. *Journal of Korean Academy of Nursing* 44(5), 471–483. doi:10.4040/jkan.2014.44.5.471 25381778

[ref42] Paiva CE, Manfredini LL, Paiva BSR, et al. (2015) The Brazilian version of the Edmonton Symptom Assessment System (ESAS) is a feasible, valid and reliable instrument for the measurement of symptoms in advanced cancer patients. *PLoS ONE* 10(7), e0132073. doi:10.1371/journal.pone.0132073 26154288 PMC4496067

[ref43] Rabow MW (2019) Meaning and relationship-centered care: recommendations for clinicians attending to the spiritual distress of patients at the end of life. *Ethics, Medicine and Public Health* 9, 57–62. doi:10.1016/j.jemep.2019.04.012

[ref44] Ramos TH, L Silva L dos S, Carvalho TP de, et al. (2024) Esperança da Pessoa com Câncer Avançado em Cuidados Paliativos. *Revista Brasileira de Cancerologia* 70(2), e-074661. doi: 10.32635/2176-9745.RBC.2024v70n2.4661

[ref45] Rizvi MAK and Hossain MZ (2017) Relationship between religious belief and happiness: A systematic literature review. *Journal of Religion and Health* 56(5), 1561–1582. doi:10.1007/s10943-016-0332-6 27909930

[ref46] Robinson L, S J and S M (2019) Cope with a life-threatening illness or serious health event. https://www.helpguide.org/wellness/health-conditions/coping-with-a-life-threatening-illness (accessed 13 October 2024).

[ref47] Rodriguez-Gonzalez A, Velasco-Durantez V, Martin-Abreu C, et al. (2022) Fatigue, emotional distress, and illness uncertainty in patients with metastatic cancer: Results from the prospective NEOETIC_SEOM study. *Current Oncology* 29(12), 9722–9732. doi:10.3390/curroncol29120763 36547177 PMC9777295

[ref48] Rudilla D, Galiana L, Oliver A, et al. (2016) Comparing counseling and dignity therapies in home care patients: a pilot study. *Palliative and Supportive Care* 14(4), 321–329. doi:10.1017/S1478951515001182 26463012

[ref49] Scarton L, Oh S, Sylvera A ,et al. (2018) Dignity Impact as a Primary Outcome Measure for Dignity Therapy. *The American Journal of Hospice & Palliative Care* 35(11), 1417–1420. doi: 10.1177/1049909118777987 29793345 PMC6082719

[ref51] Siddall P, Lovell M and MacLeod R (2015) Spirituality: what is its role in pain medicine? *Pain Medicine (Malden, Mass.)* 16(1), 51–60. doi:10.1111/pme.12511 25159525

[ref52] Silveira DR and Gradim FJ (2015) Contribuições de Viktor Frankl ao movimento da saúde coletiva. *Revista da Abordagem Gestáltica* 21(2), 153–161.

[ref53] Verkissen MN, Hjermstad MJ, Van Belle S, et al. (2019) Quality of life and symptom intensity over time in people with cancer receiving palliative care: results from the international European Palliative Care Cancer Symptom study. *PLoS ONE* 14(10), e0222988. doi:10.1371/journal.pone.0222988 31596849 PMC6784977

[ref54] Vincenzo FD, Lombardo L, Iani L, et al. (2023) Spiritual well-being, dignity-related distress and demoralisation at the end of life-effects of dignity therapy: a randomised controlled trial. *BMJ Supportive & Palliative Care* 13(e3), e1238–e1248. doi:10.1136/spcare-2022-003696 36702519

[ref55] WHO (2020) Palliative care. https://www.who.int/news-room/fact-sheets/detail/palliative-care (accessed 13 October 2024).

[ref56] Winger JG, Keeler CE and Keefe FJ (2021) Behavioural and psychosocial interventions for pain management. In *Oxford Textbook of Palliative Care*. 6th edn. Oxford University Press: Oxford, 461–470.

[ref57] Zaki-Nejad M, Nikbakht-Nasrabadi A, Manookian A, et al. (2020) The Effect of Dignity Therapy on the Quality of Life of Patients with Cancer Receiving Palliative Care. *Iranian Journal of Nursing and Midwifery Research* 25(4), 286–290. doi: 10.4103/ijnmr.IJNMR_51_19 33014739 PMC7494160

