# Supplementary material for: Effect of dignity therapy on meaning in life scores of cancer patients in palliative care
Source: Palliat Support Care. 2025 Sep 12;23:e170. doi: 10.1017/S147895152510045X (PMC13166261; doi:10.1017/S147895152510045X)
Supplement: Uchida Miwa et al. supplementary material [file S147895152510045Xsup001.docx]

**Supplementary Material**

Uchida Miwa M, Paiva CE, Ferreira ANS, Julião M, Chochinov HM, Hirai WY, Reis R, Paiva BSR. Effect of Dignity Therapy on Meaning in Life Scores of Cancer Patients in Palliative Care.

This supplementary material has been provided by the authors to give readers aditional information about their work.

**Table 1:** Factors associated with meaning in life values subscore (mean)

**Table 2:** Factors associated with meaning in life purposes subscore (mean)

**Table 3:** Factors associated with meaning in life goals subscore (mean)

**Table 4:** Factors associated with meaning in life reflections subscore (mean)

**Table 5:** Factors associated with meaning in life values subscore (pre and post-intervention)

**Table 6:** Factors associated with meaning in life purposes subscore (pre and post-intervention)

**Table 7:** Factors associated with meaning in life goals subscore (pre and post-intervention)

**Table 8:** Factors associated with meaning in life reflections subscore (pre and post-intervention)

**Table 1 -** Factors associated with meaning in life values subscore (mean)

|  | Meaning in Life Scale Values Subscore (Mean) | | | |
| --- | --- | --- | --- | --- |
|  | **Univariate estimate (95%CI)** | **p-value** | **Multivariate estimate (95%CI)** | **p-value** |
| Emotional domain^1^ | -0.05(-0.12, 0.03) | 0.2 | 0.12(-0.05, 0.29) | 0.2 |
| Physical domain^2^ | -0.02(-0.05, 0.01) | 0.2 | 0.06(-0.05, 0.17) | 0.2 |
| Spiritual domain^3^ | -0.05(-0.13, 0.03) | 0.2 | - | - |
| Total domain^4^ | -0.01(-0.03, 0.0) | 0.13 | -0.07(-0.16, 0.02) | 0.12 |
| Age | 0.01(-0.01, 0.04) | 0.3 | -0.03(-0.06, 0.0) | 0.067 |
| Gender |  |  |  |  |
| Female | - | - | - | - |
| Male | -0.03(-0.83, 0.76) | >0.9 | - | **-** |
| Religion |  |  |  |  |
| Catholic | - | - | - | - |
| Spiritist | 0.78(-0.13, 1.7) | 0.089 | - | - |
| Evangelical | 0.37(-0.46, 1.2) | 0.4 | - | - |
| Education |  |  |  |  |
| Primary | - | - | - | - |
| Secondary | -0.14(-1.0, 0.76) | 0.7 | - | - |
| Higher | -0.11(-1.1, 0.88) | 0.8 | - | - |
| Marital status |  |  |  |  |
| Married/Stable union | - | - | - | - |
| Divorced | -0.46(-1.3, 0.4) | 0.3 | -0.24(-1.2, 0.71) | 0.6 |
| Single | -1.0(-1.8, -0.26) | **0.01** | -1.0(-2.0, -0.02) | **0.046** |
| Widower | 0.84(-0.4, 2.1) | 0.2 | 1.9(0.35, 3.5) | **0.019** |
| Time since diagnosis (years) |  |  |  |  |
| < 1 | - | - | - | - |
| 1 a 5 | -0.35(-1.2, 0.47) | 0.4 | -0.7(-1.5, 0.13) | 0.094 |
| > 6 | -0.75(-1.7, 0.22) | 0.12 | -1.4(-2.5, -0.3) | **0.016** |
| PPS |  |  |  |  |
| 30% | - | - | - | - |
| 40% | -0.64(-2.2, 0.94) | 0.4 | -0.19(-1.7, 1.3) | 0.8 |
| 60% - 50% | -0.65(-2.1, 0.83) | 0.4 | -0.88(-2.2, 0.48) | 0.2 |
| 80% - 70% | -0.63(-2.3, 1.1) | 0.5 | -0.82(-2.5, 0.84) | 0.3 |

_____________________________________________________________________

PPS: Palliative Performance Scale; ^1^Emotional domain: depression and anxiety; CI: Confidence Interval; ^2^Physical domain: pain, fatigue, náusea, drowsiness, apetite, dyspnea, sleep; ^3^Espiritual domain: spiritual pain and inner peace; ^4^Total:emotional domain, physical domain, spiritual domain and wellbeing.

**Table 2 -** Factors associated with meaning in life purposes subscore (mean)

|  | Meaning in Life Scale Purposes Subscore (Mean) | | | |
| --- | --- | --- | --- | --- |
|  | **Univariate estimate (95%CI)** | **p-value** | **Multivariate estimate (95%CI)** | **p-value** |
| Emotional domain^1^ | -0.06(-0.21, 0.08) | 0.4 | 0.37(0.14, 0.6) | **0.003** |
| Physical domain^2^ | -0.05(0.1, 0.0) | 0.065 | 0.28(0.12, 0.45) | **0.002** |
| Spiritual domain^3^ | -0.24(-0.36, -0.11) | **<0.001** | - | - |
| Total domain^4^ | -0.04(-0.07, -0.01) | **0.016** | -0.25(-0.37, -0.12) | **<0.001** |
| Age | 0.04(-0.01, 0.09) | 0.1 | - | - |
| Gender |  |  |  |  |
| Female | - | - | - | - |
| Male | -0.46(-1.9, 1.0) | 0.5 | - | **-** |
| Religion |  |  |  |  |
| Catholic | - | - | - | - |
| Spiritist | 1.7(0.14, 3.3) | **0.034** | - | **-** |
| Evangelical | 1.3(-0.17, 2.7) | 0.082 | - | - |
| Education |  |  |  |  |
| Primary | - | - | - | - |
| Secondary | -1.1(-2.6, 0.36) | 0.13 | - | **-** |
| Higher | -2.2(-3.9, -0.61) | **0.009** | - | - |
| Marital status |  |  |  |  |
| Married/Stable union | - | - | - | - |
| Divorced | -0.49(-2.2, 1.2) | 0.6 | -0.21(-1.6, 1.2) | 0.8 |
| Single | -1.8(-3.3, -0.3) | **0.02** | -1.0(-2.5, 0.41) | 0.2 |
| Widower | 0.91(-1.6, 3.4) | 0.5 | 1.8(-0.27, 4.0) | 0.084 |
| Time since diagnosis (years) |  |  |  |  |
| < 1 | - | - | - | - |
| 1 a 5 | -0.12(-1.7, 1.5) | 0.9 | - | **-** |
| > 6 | -0.18(-2.1, 1.7) | 0.8 | - | - |
| PPS |  |  |  |  |
| 30% | - | - | - | - |
| 40% | -1.9(-4.6, 0.79) | 0.2 | - | **-** |
| 60% - 50% | -0.82(-3.4, 1.7) | 0.5 | - | - |
| 80% - 70% | 0.5(-2.4, 3.4) | 0.7 | - | **-** |

_____________________________________________________________________

PPS: Palliative Performance Scale; ^1^Emotional domain: depression and anxiety; CI: Confidence Interval; ^2^Physical domain: pain, fatigue, náusea, drowsiness, apetite, dyspnea, sleep; ^3^Espiritual domain: spiritual pain and inner peace; ^4^Total:emotional domain, physical domain, spiritual domain and wellbeing.

**Table 3 -** Factors associated with meaning in life goals subscore (mean)

|  | Meaning in Life Scale Goals Subscore (Mean) | | | |
| --- | --- | --- | --- | --- |
|  | **Univariate estimate (95%CI)** | **p-value** | **Multivariate estimate (95%CI)** | **p-value** |
| Emotional domain^1^ | 0.01(-0.11, 0.12) | >0.9 | 0.66(0.3, 1.0) | **<0.001** |
| Physical domain^2^ | -0.01(-0.05, 0.04) | 0.7 | 0.49(0.19, 0.8) | **0.003** |
| Spiritual domain^3^ | -0.13(-0.25, -0.02) | **0.02** | 0.26(-0.1, 0.62) | 0.15 |
| Total domain^4^ | -0.01(-0.04, 0.01) | 0.3 | -0.45(-0.74, -0.16) | **0.004** |
| Age | 0.0(-0.04, 0.04) | >0.9 | - | - |
| Gender |  |  |  |  |
| Female | - | - | - | - |
| Male | -0.84(-2.0, 0.3) | 0.14 | -0.81(-1.7, 0.07) | 0.07 |
| Religion |  |  |  |  |
| Catholic | - | - | - | - |
| Spiritist | 0.73(-0.62, 2.1) | 0.3 | - | - |
| Evangelical | 1.0(-0.2, 2.3) | 0.1 | - | - |
| Education |  |  |  |  |
| Primary | - | - | - | - |
| Secondary | -0.32(-1.7, 1.0) | 0.6 | - | - |
| Higher | -0.52(-2.0, 0.93) | 0.5 | - | - |
| Marital status |  |  |  |  |
| Married/Stable union | - | - | - | - |
| Divorced | -0.18(-1.7, 1.3) | 0.8 | - | - |
| Single | -0.64(-2.0, 0.68) | 0.3 | - | - |
| Widower | -0.78(-3.0, 1.4) | 0.5 | - | - |
| Time since diagnosis (years) |  |  |  |  |
| < 1 | - | - | - | - |
| 1 a 5 | -0.03(-1.3, 1.2) | >0.9 | 0.13(-0.81, 1.1) | 0.8 |
| > 6 | -0.27(-1.8, 1.2) | 0.7 | -1.3(-2.5, -0.09) | **0.036** |
| PPS |  |  |  |  |
| 30% | - | - | - | - |
| 40% | -0.29(-2.6, 2.0) | 0.8 | - | - |
| 60% - 50% | -0.03(-2.2, 2.1) | >0.9 | - | - |
| 80% - 70% | 1.0(-1.5, 3.5) | 0.4 | - | - |

_____________________________________________________________________

PPS: Palliative Performance Scale; ^1^Emotional domain: depression and anxiety; CI: Confidence Interval; ^2^Physical domain: pain, fatigue, náusea, drowsiness, apetite, dyspnea, sleep; ^3^Espiritual domain: spiritual pain and inner peace; ^4^Total:emotional domain, physical domain, spiritual domain and wellbeing.

**Table 4 -** Factors associated with meaning in life reflections subscore (mean)

|  | Meaning in Life Scale Reflections Subscore (Mean) | | | |
| --- | --- | --- | --- | --- |
|  | **Univariate estimate (95%CI)** | **p-value** | **Multivariate estimate (95%CI)** | **p-value** |
| Emotional domain^1^ | 0.01(-0.11, 0.14) | 0.8 | - | - |
| Physical domain^2^ | -0.04(-0.08, 0.01) | 0.086 | - | - |
| Spiritual domain^3^ | -0.05(-0.19, 0.08) | 0.4 | - | - |
| Total domain^4^ | -0.02(-0.05, 0.01) | 0.2 | - | - |
| Age | 0.03(-0.01, 0.08) | 0.11 | 0.05(0.0, 0.1) | **0.038** |
| Gender |  |  |  |  |
| Female | - | - | - | - |
| Male | -0.44(-1.7, 0.83) | 0.5 | - | - |
| Religion |  |  |  |  |
| Catholic | - | - | - | - |
| Spiritist | -0.03(-1.5, 1.5) | >0.9 | -0.11(-1.6, 1.3) | 0.9 |
| Evangelical | 0.74(-0.62, 2.1) | 0.3 | 1.6(0.01, 3.1) | **0.049** |
| Education |  |  |  |  |
| Primary | - | - | - | - |
| Secondary | -1.2(-2.6, 0.17) | 0.083 | -0.27(-1.9, 1.3) | 0.7 |
| Higher | -1.1(-2.6, 0.37) | 0.13 | 1.3(-0.9, 3.5) | 0.2 |
| Marital status |  |  |  |  |
| Married/Stable union | - | - | - | - |
| Divorced | -0.35(-1.9, 1.2) | 0.7 | - | - |
| Single | -1.1(-2.5, 0.28) | 0.11 | - | - |
| Widower | 0.25(-2.1, 2.6) | 0.8 | - | - |
| Time since diagnosis (years) |  |  |  |  |
| < 1 | - | - | - | - |
| 1 a 5 | -0.25(-1.6, 1.1) | 0.7 | - | - |
| > 6 | -0.47(-2.1, 1.2) | 0.6 | - | - |
| PPS |  |  |  |  |
| 30% | - | - | - | - |
| 40% | -1.4(-3.9, 1.0) | 0.2 | -3.0(.5.6, -0.35) | **0.028** |
| 60% - 50% | -0.26(-2.5, 2.0) | 0.8 | -1.2(-3.4, 1.1) | 0.3 |
| 80% - 70% | -0.5(-3.1, 2.1) | 0.7 | -1.8(-4.5, 0.85) | 0.2 |

_____________________________________________________________________

PPS: Palliative Performance Scale; ^1^Emotional domain: depression and anxiety; CI: Confidence Interval; ^2^Physical domain: pain, fatigue, náusea, drowsiness, apetite, dyspnea, sleep; ^3^Espiritual domain: spiritual pain and inner peace; ^4^Total:emotional domain, physical domain, spiritual domain and wellbeing.

| Table 5: Factors associated with meaning in life values subscore (pre and post-intervention) | | | | | | | | |
| --- | --- | --- | --- | --- | --- | --- | --- | --- |
| Meaning in Life Scale Values Subscore (Pre and Post Intervention)) | | | | | | | | |
|  | **Pre-intervention** | | | | **Post-intervention** | | | |
|  | **Univariate estimate (95%CI)** | **p-value** | **Multivariate estimate (95%CI)** | **p-value** | **Univariate estimate (95%CI)** | **p-value** | **Multivariate estimate (95%CI)** | **p-value** |
| Emotional domain^1^ | -0.09(-0.17, -0.02) | **0.015** | 0.19(-0.08, 0.46) | 0.2 | 0.02(-0.04, 0.08) | 0.6 | - | - |
| Physical domain^2^ | -0.02(-0.06, 0.01) | 0.2 | 0.25(0.0, 0.49) | **0.048** | -0.01(-0.03, 0.01) | 0.4 | -0.05(-0.12, 0.02) | 0.15 |
| Spiritual domain^3^ | -0.03(-0.11, 0.05) | 0.5 | 0.3(0.01, 0.59) | **0.043** | -0.05(-0.11, 0.02) | 0.13 | -0.11(-0.22, 0.0) | **0.049** |
| Total domain^4^ | -0.02(-0.04, 0.0) | 0.082 | -0.25(-0.47, -0.02) | **0.036** | -0.01(-0.02, 0.01) | 0.4 | 0.05(-0.01, 0.1) | 0.085 |
| Age | 0.02(-0.01, 0.06) | 0.2 | -0.04(-0.09, 0.01) | 0.078 | 0.01(-0.02, 0.03) | 0.6 | -0.02(-0.04, 0.01) | 0.2 |
| Gender |  |  |  |  |  |  |  |  |
| Female | - | - | - | - | - | - | - | - |
| Male | 0.32(-0.77, 1.4) | 0.6 | - | - | -0.39(-1.1, 0.29) | 0.3 | -0.44(-1.2, 0.3) | 0.2 |
| Religion |  |  |  |  |  |  |  |  |
| Catholic | - | - | - | - | - | - | - | - |
| Spiritist | 0.8(-0.5, 2.1) | 0.2 | - | - | 0.77(-0.01, 1.5) | 0.053 | - | - |
| Evangelical | 0.22(-0.96, 1.4) | 0.7 | - | - | 0.52(-0.19, 1.2) | 0.14 | - | - |
| Education |  |  |  |  |  |  |  |  |
| Primary | - | - | - | - | - | - | - | - |
| Secondary | -0.21(-1.5, 1.0) | 0.7 | -0.87(-2.0, 0.23) | 0.11 | -0.07(-0.86, 0.72) | 0.9 | - | - |
| Higher | 0.03(-1.3, 1.4) | >0.9 | -0.9(-2.4, 0.58) | 0.2 | -0.25(-1.1, 0.61) | 0.5 | - | - |
| Marital status |  |  |  |  |  |  |  |  |
| Married/Stable union | - | - | - | - | - | - | - | - |
| Divorced | 0.73(-2.0, 0.51) | 0.2 | -1.4(-2.6, -0.24) | **0.022** | -0.19(-0.96, 0.59) | 0.6 | -0.22(-1.1, 0.69) | 0.6 |
| Single | -1.3(-2.4, -0.17) | **0.025** | -1.5(-2.6, -0.37) | **0.013** | -0.76(-1.4, -0.07) | **0.032** | -0.99(-1.8, -0.17) | **0.02** |
| Widower | 0.88(-0.93, 2.7) | 0.3 | 1.1(-0.87, 3.1) | 0.2 | 0.81(-0.32, 1.9) | 0.2 | 0.96(-0.37, 2.3) | 0.15 |
| Time since diagnosis (years) |  |  |  |  |  |  |  |  |
| < 1 | - | - | - | - | - | - | - | - |
| 1 a 5 | -0.38(-1.5, 0.73) | 0.5 | -0.5(-1.7, 0.37) | 0.2 | -0.32(-1.1, 0.43) | 0.4 | -0.6(-1.3, 0.11) | 0.092 |
| > 6 | -1.3(-2.6, 0.06) | 0.061 | -1.8(-3.1, -0.61) | **0.007** | -0.25(-1.1, 0.63) | 0.6 | -0.72(-1.6, 0.18) | 0.11 |
| PPS |  |  |  |  |  |  |  |  |
| 30% | - | - | - | - | - | - | - | - |
| 40% | -1.0(-3.1, 1.1) | 0.3 | 0.11(-1.9, 2.1) | >0.9 | -0.29(-1.7, 1.1) | 0.7 | - | - |
| 60% - 50% | -1.4(-3.4, 0.57) | 0.2 | -1.3(-3.1, 0.47) | 0.13 | 0.12(-1.2, 1.4) | 0.9 | - | - |
| 80% - 70% | -1.5(-3.8, 0.79) | 0.2 | -0.61(-2.9, 1.7) | 0.6 | 0.25(-1.2, 1.7) | 0.7 | - | - |

__________________________________________________________________________________________________________________

PPS: Palliative Performance Scale; ^1^Emotional domain: depression and anxiety; CI: Confidence Interval; ^2^Physical domain: pain, fatigue, náusea, drowsiness, apetite, dyspnea, sleep; ^3^Espiritual domain: spiritual pain and inner peace; ^4^Total:emotional domain, physical domain, spiritual domain and wellbeing.

| Table 6: Factors associated with meaning in life purposes subscore (pre and post-intervention) | | | | | | | | |
| --- | --- | --- | --- | --- | --- | --- | --- | --- |
| Meaning in Life Scale Purposes Subscore (Pre and Post Intervention)) | | | | | | | | |
|  | **Pre-intervention** | | | | **Post-intervention** | | | |
|  | **Univariate estimate (95%CI)** | **p-value** | **Multivariate estimate (95%CI)** | **p-value** | **Univariate estimate (95%CI)** | **p-value** | **Multivariate estimate (95%CI)** | **p-value** |
| Emotional domain^1^ | -0.05(-0.17, 0.06) | 0.3 | 0.35(-0.05, 0.75) | 0.087 | -0.01(-0.16, 0.13) | 0.9 | 0.14(0.0, 0.28) | **0.046** |
| Physical domain^2^ | -0.03(-0.08, 0.02) | 0.2 | 0.35(-0.02, 0.73) | 0.064 | -0.05(-0.1, -0.01) | **0.026** | 0.03(-0.02, 0.09) | 0.3 |
| Spiritual domain^3^ | -0.12(-0.22, -0.02) | **0.022** | 0.26(-0.19, 0.71) | 0.2 | -0.23(-0.35, -0.1) | **<0.001** | -0.27(-0.43, -0.12) | **0.002** |
| Total domain^4^ | -0.03(-0.05, 0.0) | 0.053 | -0.32(-0.68, 0.03) | 0.073 | -0.04(-0.07, -0.01) | **0.016** | - | - |
| Age | 0.04(-0.01, 0.09) | 0.14 | - | - | 0.04(-0.01, 0.1) | 0.089 | - | - |
| Gender |  |  |  |  |  |  |  |  |
| Female | - | - | - | - | - | - | - | - |
| Male | -0.35(-1.9, 1.2) | 0.6 | - | - | -0.57(-2.1, 1.0) | 0.5 | -0.8(-2.1, 0.46) | 0.2 |
| Religion |  |  |  |  |  |  |  |  |
| Catholic | - | - | - | - | - | - | - | - |
| Spiritist | 1.8(0.18, 3.4) | **0.031** | 1.5(-0.23, 3.3) | 0.085 | 1.6(-0.07, 3.3) | 0.06 | - | - |
| Evangelical | 1.1(-0.33, 2.6) | 0.12 | 1.5(-0.13, 3.1) | 0.07 | 1.4(-0.16, 2.9) | 0.077 | - | - |
| Education |  |  |  |  |  |  |  |  |
| Primary | - | - | - | - | - | - | - | - |
| Secondary | -1.0(-2.6, 0.58) | 0.2 | - | - | -1.3(-2.9, 0.3) | 0.11 | 0.1(-1.5, 1.7) | >0.9 |
| Higher | -2.0(-3.8, -0.33) | **0.021** | - | - | -2.4(-4.2, -0.72) | 0.007 | -0.78(-2.6, 1.0) | 0.4 |
| Marital status |  |  |  |  |  |  |  |  |
| Married/Stable union | - | - | - | - | - | - | - | - |
| Divorced | -0.66(-2.4, 1.1) | 0.4 | -1.6(-3.4, 0.17) | 0.074 | -0.33(-2.1, 1.5) | 0.7 | 0.12(-1.6, 1.8) | 0.9 |
| Single | -1.6(-3.2, -0.06) | **0.042** | -0.68(-2.3, 0.9) | 0.4 | -2.0(-3.6, -0.4) | **0.016** | -1.8(-3.2, -0.34) | **0.019** |
| Widower | 0.94(-1.7, 3.5) | 0.5 | 0.5(-2.3, 3.3) | 0.7 | 0.88(-1.8, 3.5) | 0.5 | 0.42(-1.7, 2.6) | 0.7 |
| Time since diagnosis (years) |  |  |  |  |  |  |  |  |
| < 1 | - | - | - | - | - | - | - | - |
| 1 a 5 | -0.08(-1.7, 1.6) | >0.9 | - | - | -0.15(-1.9, 1.6) | 0.9 | -0.23(-1.4, 0.95) | 0.7 |
| > 6 | -0.32(-2.3, 1.6) | 0.7 | - | - | -0.04(-2.1, 2.0) | >0.9 | -1.4(-2.9, 0.08) | 0.061 |
| PPS |  |  |  |  |  |  |  |  |
| 30% | - | - | - | - | - | - | - | - |
| 40% | -1.8(-4.7, 1.1) | 0.2 | - | - | -2.1(-4.9, 0.76) | 0.14 | -2.0(-4.3, 0.26) | 0.078 |
| 60% - 50% | -0.85(-3.5, 1.8) | 0.5 | - | - | -0.79(-3.4, 1.8) | 0.5 | -2.0(-4.0, 0.12) | 0.063 |
| 80% - 70% | 0.25(-2.8, 3.3) | 0.9 | - | - | 0.75(-2.3, 3.8) | 0.6 | 0.15(-2.4, 2.7) | >0.9 |

__________________________________________________________________________________________________________________

PPS: Palliative Performance Scale; ^1^Emotional domain: depression and anxiety; CI: Confidence Interval; ^2^Physical domain: pain, fatigue, náusea, drowsiness, apetite, dyspnea, sleep; ^3^Espiritual domain: spiritual pain and inner peace; ^4^Total:emotional domain, physical domain, spiritual domain and wellbeing.

| Table 7: Factors associated with meaning in life goals subscore (pre and post-intervention) | | | | | | | | |
| --- | --- | --- | --- | --- | --- | --- | --- | --- |
| Meaning in Life Scale Goals Subscore (Pre and Post Intervention)) | | | | | | | | |
|  | **Pre-intervention** | | | | **Post-intervention** | | | |
|  | **Univariate estimate (95%CI)** | **p-value** | **Multivariate estimate (95%CI)** | **p-value** | **Univariate estimate (95%CI)** | **p-value** | **Multivariate estimate (95%CI)** | **p-value** |
| Emotional domain^1^ | 0.0(-0.1, 0.1) | >0,9 | 0.37(-0.16, 0.89) | 0.2 | 0.0(-0.11, 0.1) | >0.9 | 0.34(0.15, 0.52) | **0.002** |
| Physical domain^2^ | 0.0(-0.05, 0.04) | >0.9 | 0.49(0.02, 0.95) | **0.043** | -0.01(-0.05, 0.03) | 0.6 | 0.26(0.12, 0.39) | **0.001** |
| Spiritual domain^3^ | -0.05(-0.15, 0.05) | 0.3 | 0.45(-0.1, 1.0) | 0.1 | -0.12(-0.23, -0.02) | **0.023** | - | - |
| Total domain^4^ | -0.01(-0.03, 0.02) | 0.6 | -0.42(-0.85, 0.02) | 0.058 | -0.01(-0.04, 0.01) | 0.3 | -0.19(-0.29, -0.08) | **0.002** |
| Age | -0.01(-0.05, 0.04) | 0.8 | -0.04(-0.13, 0.04) | 0.3 | 0.01(-0.03, 0.05) | 0.7 | 0.03(-0.01, 0.08) | 0.2 |
| Gender |  |  |  |  |  |  |  |  |
| Female | - | - | - | - | - | - | - | - |
| Male | -0.51(-1.9, 0.84) | 0.4 | -0.84(-2.7, 1.0) | 0.3 | -1.2(-2.3, -0.06) | **0.04** | -1.8(-2.9, -0.7) | **0.004** |
| Religion |  |  |  |  |  |  |  |  |
| Catholic | - | - | - | - | - | - | - | - |
| Spiritist | 1.0(-0.58, 2.6) | 0.2 | - | - | 0.47(-0.9, 1.8) | 0.5 | - | - |
| Evangelical | 1.0(-0.43, 2.4) | 0.2 | - | - | 1.1(-0.19, 2.3) | 0.093 | - | - |
| Education |  |  |  |  |  |  |  |  |
| Primary | - | - | - | - | - | - | - | - |
| Secondary | -0.86(-2.4, 0.67) | 0.3 | -1.5(-3.6, 0.63) | 0.2 | 0.21(-1.1, 1.6 | 0.7 | 1.4(0.03, 2.7) | **0.046** |
| Higher | -0.81(-2.5, 0.85) | 0.3 | -2.3(-5.2, 0.59) | 0.11 | -0.24(-1.7, 1.2) | 0.7 | 0.48(-1.2, 2.2) | 0.5 |
| Marital status |  |  |  |  |  |  |  |  |
| Married/Stable union | - | - | - | - | - | - | - | - |
| Divorced | -0.21(-1.9, 1.5) | 0.8 | -2.1(-4.5, 0.35) | 0.087 | -0.15(-1.7, 1.4) | 0.8 | -0.62(-2.0, 0.79) | 0.4 |
| Single | -0.96(-2.5, 0.56) | 0.2 | -1.8(-3.9, 0.37) | 0.1 | -0.32(-1.7, 1.0) | 0.6 | -0.96(-2.2, 0.28) | 0.12 |
| Widower | -0.81(-3.3, 1.7) | 0.5 | -2.1(-6.1, 1.9) | 0.3 | -0.75(-3.0, 1.5) | 0.5 | -2.4(-4.4, -0.43) | **0.021** |
| Time since diagnosis (years) |  |  |  |  |  |  |  |  |
| < 1 | - | - | - | - | - | - | - | - |
| 1 a 5 | 0.29(-1.2, 1.8) | 0.7 | 0.44(-1.5, 2.4) | 0.6 | -0.34(-1.6, 0.93) | 0.6 | 0.47(-0.62, 1.6) | 0.4 |
| > 6 | -0.09(-1.8, 1.7) | >0.9 | -1.1(-3.4, 1.3) | 0.3 | -0.45(-2.0, 1.1) | 0.5 | -0.63(-2.0, 0.76) | 0.3 |
| PPS |  |  |  |  |  |  |  |  |
| 30% | - | - | - | - | - | - | - | - |
| 40% | -0.36(-3.0, 2.3) | 0.8 | 2.3(-1.5, 6.1) | 0.2 | -0.21(-2.5, 2.1) | 0.9 | -0.73(-2.7, 1.2) | 0.4 |
| 60% - 50% | -0.09(-2.6, 2.4) | >0.9 | 1.7(-1.8, 5.1) | 0.3 | 0.03(-2.1, 2.2) | >0.9 | -0.24(-2.0, 1.5) | 0.8 |
| 80% - 70% | 1.0(-1.9, 3.9) | 0.5 | 3.6(-0.79, 8.0) | 0.1 | 1.0(-1.5, 3.5) | 0.4 | 1.1(-1.0, 3.2) | 0.3 |

__________________________________________________________________________________________________________________

PPS: Palliative Performance Scale; ^1^Emotional domain: depression and anxiety; CI: Confidence Interval; ^2^Physical domain: pain, fatigue, náusea, drowsiness, apetite, dyspnea, sleep; ^3^Espiritual domain: spiritual pain and inner peace; ^4^Total:emotional domain, physical domain, spiritual domain and wellbeing.

| Table 8: Factors associated with meaning in life reflections subscore (pre and post-intervention) | | | | | | | | |
| --- | --- | --- | --- | --- | --- | --- | --- | --- |
| Meaning in Life Scale Reflections Subscore (Pre and Post Intervention)) | | | | | | | | |
|  | **Pre-intervention** | | | | **Post-intervention** | | | |
|  | **Univariate estimate (95%CI)** | **p-value** | **Multivariate estimate (95%CI)** | **p-value** | **Univariate estimate (95%CI)** | **p-value** | **Multivariate estimate (95%CI)** | **p-value** |
| Emotional domain^1^ | 0.01(-0.09, 0.11) | 0.8 | - | - | 0.01(-0.12, 0.13) | >0.9 | - | - |
| Physical domain^2^ | -0.02(-0.07, 0.02) | 0.3 | - | - | -0.03(-0.07, 0.01) | 0.2 | -0.03(-0.08, 0.02) | 0.2 |
| Spiritual domain^3^ | -0.02(-0.12, 0.08) | 0.7 | - | - | -0.04(-0.17, 0.09) | 0.5 | - | - |
| Total domain^4^ | -0.01(-0.03, 0.02) | 0.4 | - | - | -0.01(-0.04, 0.01) | 0.3 | - | - |
| Age | 0.03(-0.02, 0.07) | 0.2 | 0.04(-0.01, 0.1) | 0.1 | 0.04(-0.01, 0.08) | 0.084 | 0.06(0.0, 0.11) | **0.044** |
| Gender |  |  |  |  |  |  |  |  |
| Female | - | - | - | - | - | - | - | - |
| Male | -0.33(-1.7, 1.0) | 0.6 | - | - | -0.56(-1.9, 0.8) | 0.4 | - | - |
| Religion |  |  |  |  |  |  |  |  |
| Catholic | - | - | - | - | - | - | - | - |
| Spiritist | -0.37(-1.9, 1.2) | 0.6 | -0.47(-2.0, 1.1) | 0.5 | 0.3(-1.3, 1.9) | 0.7 | 0.35(-1.2, 1.9) | 0.6 |
| Evangelical | 0.68(-0.74, 2.1) | 0.3 | 1.4(-0.29, 3.1) | 0.1 | 0.8(-0.65, 2.3) | 0.3 | 1.9(0.21, 3.6) | **0.029** |
| Education |  |  |  |  |  |  |  |  |
| Primary | - | - | - | - | - | - | - | - |
| Secondary | -1.2(-2.7, 0.24) | 0.1 | -0.41(-2.1, 1.3) | 0.6 | -1.2(-2.7, 0.27) | 0.11 | -0.34(-2.1, 1.4) | 0.7 |
| Higher | -1.2(-2.8, 0.41) | 0.14 | 1.0(-1.4, 3.4) | 0.4 | -1.1(-2.7, 0.52) | 0.2 | 1.5(-0.87, 3.8) | 0.2 |
| Marital status |  |  |  |  |  |  |  |  |
| Married/Stable union | - | - | - | - | - | - | - | - |
| Divorced | -0.43(-2.1, 1.3) | 0.6 | - | - | -0.28(-1.9, 1.4) | 0.7 | - | - |
| Single | -0.91(-2.4, 0.57) | 0.2 | - | - | -1.3(-2.8, 0.17) | 0.08 | - | - |
| Widower | 0.38(-2.1, 2.8) | 0.8 | - | - | 0.13(-2.3, 2.6) | >0.9 | - | - |
| Time since diagnosis (years) |  |  |  |  |  |  |  |  |
| < 1 | - | - | - | - | - | - | - | - |
| 1 a 5 | -0.17(-1.6, 1.3) | 0.8 | - | - | -0.34(-1.8, 1.1) | 0.6 | - | - |
| > 6 | -0.21(-1.9, 1.5) | 0.8 | - | - | -0.73(-2.5, 1.0) | 0.4 | - | - |
| PPS |  |  |  |  |  |  |  |  |
| 30% | - | - | - | - | - | - | - | - |
| 40% | -1.6(-4.1, 0.99) | 0.2 | -2.9(-5.8, -0.06) | **0.046** | -1.3(-3.9, 1.4) | 0.3 | -2.6(-5.5, 0.24) | 0.07 |
| 60% - 50% | -0.35(-2.7, 2.0) | 0.8 | -1.1(-3.6, 1.3) | 0.3 | -0.18(-2.6, 2.3) | 0.9 | -0.95(-3.4, 1.5) | 0.4 |
| 80% - 70% | -0.5(-3.3, 2.3) | 0.7 | -1.7(-4.6, 1.3) | 0.3 | -0.5(-3.4, 2.4) | 0.7 | -2.3(-5.2, 0.58) | 0.11 |

__________________________________________________________________________________________________________________

PPS: Palliative Performance Scale; ^1^Emotional domain: depression and anxiety; CI: Confidence Interval; ^2^Physical domain: pain, fatigue, náusea, drowsiness, apetite, dyspnea, sleep; ^3^Espiritual domain: spiritual pain and inner peace; ^4^Total:emotional domain, physical domain, spiritual domain and wellbeing.
